# Long-term prognostic value of inflammatory biomarkers for patients with acute heart failure: Construction of an inflammatory prognostic scoring system

**DOI:** 10.3389/fimmu.2022.1005697

**Published:** 2022-09-15

**Authors:** Xu Zhu, Iokfai Cheang, Fang Xu, Rongrong Gao, Shengen Liao, Wenming Yao, Yanli Zhou, Haifeng Zhang, Xinli Li

**Affiliations:** ^1^ Department of Cardiology, The First Affiliated Hospital of Nanjing Medical University, Jiangsu Province Hospital, Nanjing, China; ^2^ Department of Cardiology, The Affiliated Suzhou Hospital of Nanjing Medical University, Suzhou Municipal Hospital, Suzhou, China

**Keywords:** inflammation, biomarkers, inflammatory prognostic scoring (IPS), all-cause mortality, random survival forest, acute heart failure (AHF)

## Abstract

**Objective:**

Systemic inflammation is associated with a poor prognosis in acute heart failure (AHF). This study was to assess the long-term prognostic value of combining the accessible inflammatory markers in relation to all-cause mortality in patients with AHF.

**Methods:**

Consecutive patients with AHF who were hospitalized between March 2012 and April 2016 at the Department of Cardiology of the First Affiliated Hospital of Nanjing Medical University were enrolled in this prospective study. The LASSO regression model was used to select the most valuable inflammatory biomarkers to develop an inflammatory prognostic scoring (IPS) system. Kaplan-Meier method, multivariate COX regression and time-dependent ROC analysis were used to assess the relationship between inflammatory markers and AHF prognosis. A randomized survival forest model was used to estimate the relative importance of each inflammatory marker in the prognostic risks of AHF.

**Results:**

A total of 538 patients with AHF were included in the analysis (mean age, 61.1 ± 16.0 years; 357 [66.4%] men). During a median follow-up of 34 months, there were 227 all-cause deaths (42.2%). C-reactive protein (CRP), red blood cell distribution width (RDW) and neutrophil-to-lymphocyte ratio (NLR) were incorporated into the IPS system (IPS = 0.301×CRP + 0.263×RDW + 0.091×NLR). A higher IPS meant a significantly worse long-term prognosis in Kaplan-Meier analysis, with 0.301 points as the optimal cut-off value (*P* log-rank <0.001). IPS remained an independent prognostic factor associated with an increased risk of all-cause mortality among patients with AHF in multivariate Cox regression models with a full adjustment of the other significant covariables. Random forest variable importance and minimal depth analysis further validated that the IPS system was the most predictive for all-cause mortality in patients with AHF.

**Conclusions:**

Inflammatory biomarkers were associated with the risk of all-cause mortality in patients with AHF, while IPS significantly improved the predictive power of the model and could be used as a practical tool for individualized risk stratification of patients with AHF.

## Introduction

Heart failure (HF) remains a major cause of mortality worldwide, with the 5-year mortality rate approaching 50% (1). Due to population aging and advances in HF treatment, the overall prevalence of HF is approximately 1.5-4.0% and has been increasing (2–4), which causes a huge socioeconomic burden. Despite the advances and development in the treatment of HF, the hospitalization and mortality rate of HF is still high (5).

Inflammation plays a central role in the pathogenesis and progression of HF, which can promote myocardial fibrosis and remodeling through different mechanistic pathways (6). It has been recognized as a common pathobiological feature of acute HF (AHF) and chronic HF (CHF), leading to the impairment of cardiac structures and functions, which may be related to innate and humoral immune system activation, endothelial inflammation and systemic inflammatory mediators (7). Specific inflammatory biomarkers elevated in patients with HF may reflect their involvement in disease pathogenesis (8, 9). The level of these specific circulating inflammatory biomarkers has been associated with disease severity and prognosis in patients with HF independently of traditional biomarkers (7, 10). While the early diagnosis and optimization management of patients with AHF could improve the prognosis to a certain extent, and biomarkers that reflect the pathophysiological pathways of AHF development could certainly be utilized for risk assessment as well as prognostic prediction (11).

Among inflammatory markers, such as C-reactive protein (CRP), markers derived from complete blood count (including white blood cells [WBC], neutrophils [NEU], lymphocytes [LYM], monocytes [Mon], red blood cell distribution width [RDW] and platelets [PLT] have shown their important roles in inflammatory and immune responses, and changes in their level were associated with cardiovascular diseases (CVDs) as well as all-cause mortality (12–17). More recently, inflammatory parameters derived from complete blood count (CBC), including neutrophil-to-lymphocyte ratio (NLR), platelet-to-lymphocyte ratio (PLR), lymphocyte-to-monocyte ratio (LMR), systemic immune-inflammation index (SII) and systemic inflammation response index (SIRI), have been shown to be novel inflammatory biomarkers associated with cardiovascular diseases and their prognosis (18–22). These inflammatory markers are not only easy to access, but can also be used to quantitatively assess the condition of patients.

Increasing attention is being paid to the establishment of prognostic models based on the above-mentioned inflammatory biomarkers/parameters for individualized prognostic prediction in patients with AHF. However, comprehensive analyses comparing and integrating these markers in assessing the risks of all-cause mortality in patients with AHF have not been performed. We hypothesized that compared to a single biomarker, a combination of all these biomarkers might be more valuable and could provide more accurate information for survival prediction. The aim of this study was to comprehensively analyze and compare the association of CRP, CBC and their derived inflammatory biomarkers with the all-cause mortality in patients with AHF, so as to further develop a prognostic model – an inflammation prognostic scoring (IPS) system for the individualized prediction of survival probability in patients with AHF.

## Methods

### Participants and study design

**Figure 1:**
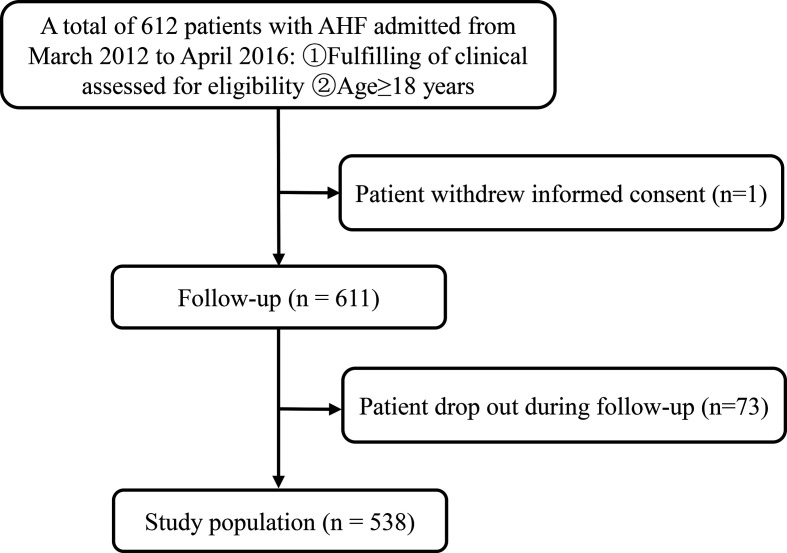
Flowchart of the study participants.

A total of 612 consecutive patients were prospectively enrolled in this study, who hospitalized for AHF in the Department of Cardiology, the First Affiliated Hospital of Nanjing Medical University from March 2012 to April 2016. Among them, 538 patients with AHF were included in further analyses (**Figure 1**). AHF refers to patients with an acute decompensation caused by chronic heart failure (ADHF) and acute new-onset HF. All participants were aged over 18 years, who were diagnosed and received standard treatment according to Chinese guidelines for the diagnosis and treatment of HF (23). Patients with malignant tumors, severe mental illnesses and/or uncontrolled systemic diseases were excluded.

The study protocols were approved by the Independent Ethics Committee (First Affiliated Hospital of Nanjing Medical University, Nanjing, Jiangsu, China) and conducted in accordance with the *Declaration of Helsinki*. Each participant had provided a signed informed consent. The trial was registered at http://www.chictr.org.cn/(Trial registration: ChiCTR - ONC-12001944, Registered 5 Feb 2012, http://www.chictr.org.cn/showprojen.aspx?proj=7604).

The primary endpoint was defined as all-cause death. During the follow-up period, patients were evaluated by telephone and/or outpatient visit once every 3 months. Endpoint events were confirmed by medical staff and patients’ families.

### Data collection

Within 24 hours of admission, the baseline characteristics of the patients were collected, including demographic characteristics, comorbidities (hypertension, diabetes mellitus, and ischemic etiology), physical examination (heart rate, blood pressure, body mass index), laboratory tests (hemoglobin, total cholesterol, triglycerides, high-density lipoprotein cholesterol, low-density lipoprotein cholesterol, serum glucose, blood urea nitrogen [BUN], estimated glomerular filtration rate [eGFR], Troponin T, N-terminal prohormone B-type Natriuretic Peptide [NT-proBNP]), transthoracic echocardiography (left ventricular ejection fraction [LVEF]) and medication therapies (diuretics, aldosterone antagonist, angiotensin converting enzyme inhibitor/angiotensin receptor blocker [ACEI/ARB], beta blockers).

All venous blood, including CBC, biochemistry panel, coagulation, thyroid functions and NT-proBNP, was analyzed in the central laboratory of the First Affiliated Hospital of Nanjing Medical University. TTE was performed using the Vivid E9 ultrasound system (GE Healthcare, USA) to assess cardiac function parameters; and Simpson’s method was used to assess LVEF. The eGFR was calculated using a formula developed by the Chronic Kidney Disease Epidemiology (CKD-EPI) Collaborative Institute (24).

### Inflammation biomarkers

CRP was detected by a Siemens BN-II specific protein analyzer and supporting reagents. CBC analysis included the WBC, NEU, LYM, Mon, RDW and PLT. Furthermore, a complete blood count was derived based on inflammatory parameters, including NLR, PLR, LMR, SII and SIRI, which were calculated as follows: 1) NLR = NEU (10^9^/L)/LYM (10^9^/L); 2) PLR = PLT (10^9^/L)/LYM (10^9^/L); 3) LMR = LYM (10^9^/L)/Mon (10^9^/L); 4) SII = NEU (10^9^/L) × PLT (10^9^/L)/LYM (10^9^/L); 5) SIRI = NEU (10^9^/L) × Mon (10^9^/L)/LYM (10^9^/L).

### Development of inflammatory prognostic scoring system

The median follow-up time of patients with AHF in this study was 34 months; therefore, 36 months was taken as the time cut-off point for prognostic assessment. A time-dependent receiver operating characteristic curve (ROC curve) was performed by the R package “timeROC” to evaluate the predictive value of CRP and CBC, of which inflammatory markers were derived from CBC for all-cause mortality in patients with AHF (25, 26). The optimal cut-off value of these 12 inflammatory markers for predicting all-cause mortality of patients with AHF was identified, which were then classified as categorical variables according to the cut-off value, respectively. Considering the possibility of multicollinearity of inflammatory biomarkers, we performed the Least Absolute Shrinkage and Selection Operator (LASSO) analysis, with 5-fold cross-validation for data dimensionality reduction and variable selection using the R package “glmnet”. Inflammatory biomarkers with non-zero coefficients in the LASSO-COX regression analysis were incorporated to construct the novel IPS, which was calculated as follows: IPS = sum (the score of every inflammatory biomarker × corresponding regression coefficients from LASSO).

### Statistical analysis

Continuous variables were expressed as mean ± standard deviations (SD) or medians (interquartile range, Q1-Q3). Categorical variables were expressed by n (%). Skewed data was log-transformed to fit a normal distribution. Continuous variables were compared between groups using unpaired t-test (normal distribution) or Mann-Whitney U test (non-normal distribution). Categorical variables were compared using Pearson’s χ test. Multiple imputation for missing data was completed using the ‘mice’ package based on the random forest algorithm. *P* value <0.05 was considered as being statistically significant. All statistical analyses were performed using SPSS 24.0 and R 4.0.3.

Pearson correlation analysis was used to calculate the matrix of correlation coefficients among 12 inflammatory markers pairwise. Survival differences between groups were compared using the Kaplan-Meier analysis and log-rank test. Multivariate COX regression analysis was used to establish the basic model for all-cause mortality in patients with AHF. Based on important clinical risk factors for cardiovascular diseases (except CRP, CBC and their derived inflammatory biomarkers), variables with *P* < 0.10 in the univariate analysis were incorporated into the model, which were screened based on conditional likelihood ratios to construct a basic model for the prognosis of patients with AHF. Time-dependent ROC (1, 3 and 5 years) was used to evaluate the improvement of inflammatory biomarkers and IPS on the basic model for all-cause mortality in patients with AHF.

The random survival forests (RSF) model developed by Breiman L (27). was used to estimate the relative importance of each inflammatory marker while predicting the risk of all-cause mortality in AHF. The rank of each variable was based on 2 predictive indicators for all-cause mortality risk: 1) minimal depth (MD), where variables that had a short MD and split the tree near the root were highly predictive; 2) variable importance (VIMP), where variables with a higher VIMP value were more predictive (28).

According to the findings of the EHFS II study (29), the incidence of death at 12 months after hospital discharge was 28.4% in older patients (median age 83.7 years) and 18.5% in younger patients (median age 68.4 years) with acute heart failure. Thus, the sample size was adjusted for an anticipated event rate of 25%. Based on a sample of 382 observations achieves 80% power at a 0.05 significance level to detect a hazard ratio (HR) of 1.5. In addition, 10% dropout rate (DR) also taken into account and the minimum sample size was 420. The sample size was calculated using PASS (Version 11).

## Results

### Baseline characteristics

A total of 538 patients with AHF were enrolled in this study. During the median follow-up of 34 months, all-cause mortality occurred on 227 (42.2%) patients. Patients were divided into death group and survival group based on the outcome, CRP, NEU, RDW, NLR, SII and SIRI were higher in the AHF death group, in which LYM, PLT and LMR were lower (All *P*<0.05). While WBC, Mon or PLR did not show difference between groups. Overall, patients with AHF in the death group were older, had more severe HF-related symptoms, a poorer nutritional status, higher NT-proBNP levels and more pronounced systemic inflammatory activation between groups (**Table 1**).

**Table 1:** Baseline characteristics in patients with AHF.

Variables	Total (n=538)	Survival (n=311)	Death (n=227)	*P* value
Age, years	61.07 (15.98)	58.03 (15.91)	65.23 (15.16)	0.005
Male, %	357 (66.4%)	228 (73.3%)	129 (56.8%)	<0.001
LVEF, %	42.11 (14.46)	41.07 (14.14)	43.54 (14.81)	0.050
**Medical history, %**				
Hypertension	275 (51.1%)	166 (53.4%)	109 (48.0%)	0.254
Diabetes mellitus	131 (24.3%)	74 (23.8%)	57 (25.1%)	0.803
Ischemic etiology	204 (37.9%)	121 (38.9%)	83 (36.6%)	0.643
**NYHA functional class, %**				0.009
II	91 (16.9%)	65 (20.9%)	26 (11.5%)	
III	289 (53.7%)	164 (52.7%)	125 (55.1%)	
IV	158 (29.4%)	82 (26.4%)	76 (33.5%)	
**Physical examination**				
Average heart rate, bpm	79.01 (15.59)	80.13 (15.31)	77.48 (15.86)	0.051
Systolic BP, mmHg	126.49 (22.07)	128.71 (23.56)	123.45 (19.49)	<0.001
Diastolic BP, mmHg	78.45 (14.99)	80.50 (16.43)	75.64 (12.23)	<0.001
MAP, mmHg	94.46 (15.56)	96.57 (16.93)	91.58 (12.94)	<0.001
BMI, kg/M^2^	24.23 (4.54)	24.44 (4.33)	23.96 (4.80)	0.228
**Laboratory measures**				
Hemoglobin, g/L	134.00 [119.25, 147.00]	136.00 [123.00, 149.00]	129.00 [116.00, 144.00]	<0.001
Total cholesterol, mmol/L	3.98 (1.04)	4.03 (0.99)	3.91 (1.11)	0.199
Triglycerides, mmol/L	1.25 (0.66)	1.33 (0.71)	1.14 (0.56)	<0.001
HDL-C, mmol/L	0.99 (0.29)	0.98 (0.27)	0.99 (0.32)	0.542
LDL-C, mmol/L	2.56 (0.86)	2.59 (0.78)	2.52 (0.95)	0.369
Serum glucose, mmol/L	5.55 (2.07)	5.44 (1.77)	5.70 (2.41)	0.150
BUN, mmol/L	17.69 (11.72)	16.01 (11.25)	19.99 (11.98)	<0.001
eGFR, mL/(min·1.73 m^2^)	72.72 (26.21)	78.00 (24.85)	65.4894 (26.35)	<0.001
Troponin T, ng/mL	0.05 [0.05, 27.46]	0.35 [0.05,31.74]	0.05 [0.05,19.40]	0.008
NT-proBNP, ng/L	2225.50 [1269.00, 5640.00]	1791.00 [1087.00,4643.50]	2777.00 [1590.00,7250.00]	<0.001
**Inflammatory biomarkers**				
C-reactive protein, mg/L	4.60 [3.44, 16.45]	3.90 [3.40, 12.00]	7.10 [3.44, 30.20]	<0.001
White blood cell count, 10^9^/L	6.60 [5.30, 8.52]	6.54 [5.30, 8.00]	6.80 [5.29, 9.00]	0.196
Neutrophils count, 10^9^/L	4.20 [3.23, 6.01]	4.19 [3.13, 5.44]	4.38 [3.40, 6.64]	0.026
Lymphocyte count, 10^9^/L	1.56 [1.14, 2.06]	1.65 [1.24, 2.12]	1.48 [1.06, 1.97]	0.002
Monocyte count, 10^9^/L	0.45 [0.33, 0.60]	0.43 [0.33, 0.58]	0.48 [0.34, 0.62]	0.143
RDW, %	14.30 [13.40, 15.60]	14.00 [13.20, 15.10]	14.80 [13.80, 16.10]	<0.001
Platelet count, 10^9^/L	164.00 [124.00, 206.00]	167.00 [134.00, 205.00]	154.00 [116.00, 209.00]	0.040
NLR	2.64 [1.82, 4.47]	2.40 [1.64, 4.04]	3.05 [2.14, 5.21]	<0.001
PLR	100.00 [75.29, 140.67]	97.73 [75.31, 134.93]	105.75 [75.78, 146.30]	0.178
LMR	3.50 [2.34, 5.00]	3.86 [2.51, 5.27]	3.09 [2.21, 4.52]	0.001
SII, 10^9^/L	427.42 [280.05, 712.04]	398.01 [257.22, 689.35]	465.68 [315.12, 776.62]	0.015
SIRI, 10^9^/L	1.19 [0.74, 2.23]	1.08 [0.64, 2.05]	1.46 [0.86, 2.55]	<0.001
**Prior medication, %**				
Diuretics	510 (94.8%)	294 (94.5%)	216 (95.2%)	0.902
Aldosterone antagonist	479 (89.0%)	279 (89.7%)	200 (88.1%)	0.654
ACEI/ARB	417 (77.5%)	248 (79.7%)	169 (74.4%)	0.178
Beta blockers	427 (79.4%)	247 (79.4%)	180 (79.3%)	0.967

Continuous variables are expressed as mean ± SD or as median [interquartile range] and categorical variables are expressed as number (%).

LVEF, left ventricular ejection fraction; NYHA, New York Heart Association; BP, blood pressure; MAP, mean arterial pressure; BMI, body mass index; HDL-C, high-density lipoprotein cholesterol; LDL-C, low-density lipoprotein cholesterol; BUN, blood urea nitrogen; eGFR, estimated glomerular filtration rate; NT-pro BNP, N-Terminal pro-brain natriuretic peptide; RDW, red blood cell distribution width; NLR, neutrophil-to-lymphocyte ratio; PLR, platelet to lymphocyte ratio; LMR, lymphocyte-to-monocyte ratio; SII, systemic inflammation index; SIRI, systemic inflammatory response index.

### Optimal cut-off of inflammatory biomarkers for predicting all-cause mortality of AHF

**Figure 2:**
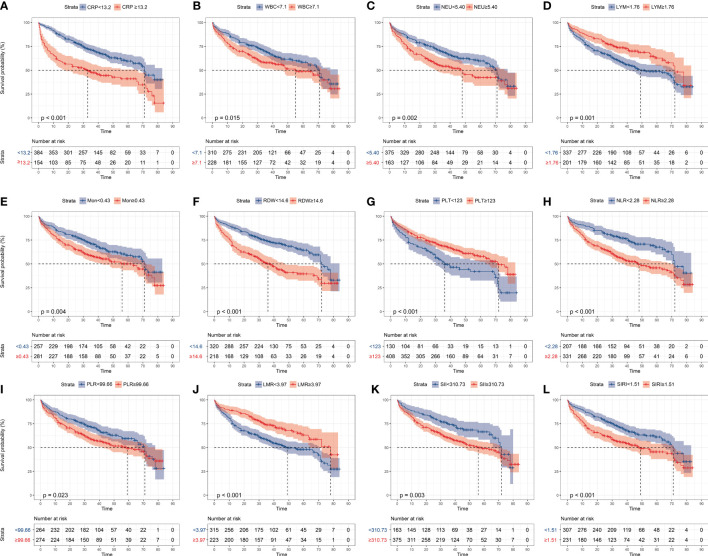
Kaplan-Meier analysis of the inflammatory markers in all-cause mortality in patients with AHF, grouped by ROC optimal cut-off values. **(A)** CRP, C-reactive protein; **(B)** WBC, white blood cell; **(C)** NEU, neutrophils; **(D)** LYM, lymphocyte; **(E)** Mon, monocyte; **(F)**: RDW, red cell distribution width; **(G)** PLT, platelet; **(H)** NLR, neutrophil-to-lymphocyte ratio; **(I)** PLR, platelet-to-lymphocyte ratio; **(J)** LMR, lymphocyte-to-monocyte ratio monocyte; **(K)** SII, systemic immune-inflammation index; **(L)** SIRI, systemic inflammatory response index.

A 3-year time-dependent ROC curve was constructed using all-cause deaths in patients with AHF as the outcome variables, and the optimal cut-off value was determined by the maximum Youden index (**Table 2**). Based on the cut-off values, patients were characterized as low- and high-inflammatory-response group. Kaplan-Meier survival curves showed that patients with AHF in the high-inflammatory-response group (CRP≥13.2 mg/L, WBC≥7.10 ×10^9^/L, NEU≥5.40 ×10^9^/L, LYM<1.76 ×10^9^/L, Mon≥0.43 ×10^9^/L, RDW≥14.6%, PLT<123 ×10^9^/L, NLR≥2.28, PLR≥99.66, LMR<3.97, SII≥310.73 ×10^9^/L, and SIRI≥1.51 ×10^9^/L) had a higher all-cause mortality (All log-rank test: *P <*0.05, **Figure 2**).

**Table 2:** Area under the curve (AUC) and optimal threshold for inflammatory biomarkers to predict 3-year all-cause mortality in patients with AHF.

	AUC (95% CI)	Cut-off value	Sensitivity	Specificity
CRP, mg/L	0.594 (0.538-0.650)	13.20	0.425	0.76
WBC, 10^9^/L	0.547 (0.490-0.604)	7.10	0.501	0.636
NEU, 10^9^/L	0.584 (0.529-0.640)	5.40	0.389	0.764
LYM, 10^9^/L	0.407 (0.352-0.462)	1.76	0.279	0.556
Mon, 10^9^/L	0.538 (0.481-0.595)	0.43	0.609	0.538
RDW, %	0.644 (0.591-0.698)	14.6	0.561	0.68
PLT, 10^9^/L	0.454 (0.396-0.511)	123	0.668	0.260
NLR	0.637 (0.584-0.690)	2.28	0.751	0.480
PLR	0.549 (0.493-0.606)	99.66	0.595	0.556
LMR	0.414 (0.358-0.442)	3.97	0.305	0.524
SII, 10^9^/L	0.571 (0.515-0.626)	310.73	0.782	0.360
SIRI, 10^9^/L	0.604 (0.549-0.659)	1.51	0.548	0.631

CRP, C-reactive protein; WBC, white blood cell; NEU, neutrophils; LYM, lymphocyte; Mon, monocyte; RDW, red blood cell distribution width; PLT, platelet; NLR, neutrophil-to-lymphocyte ratio; PLR, platelet to lymphocyte ratio; LMR, lymphocyte-to-monocyte ratio; SII, systemic inflammation index; SIRI, systemic inflammatory response index.

### Development of inflammatory prognostic scoring system

**Figure 3:**
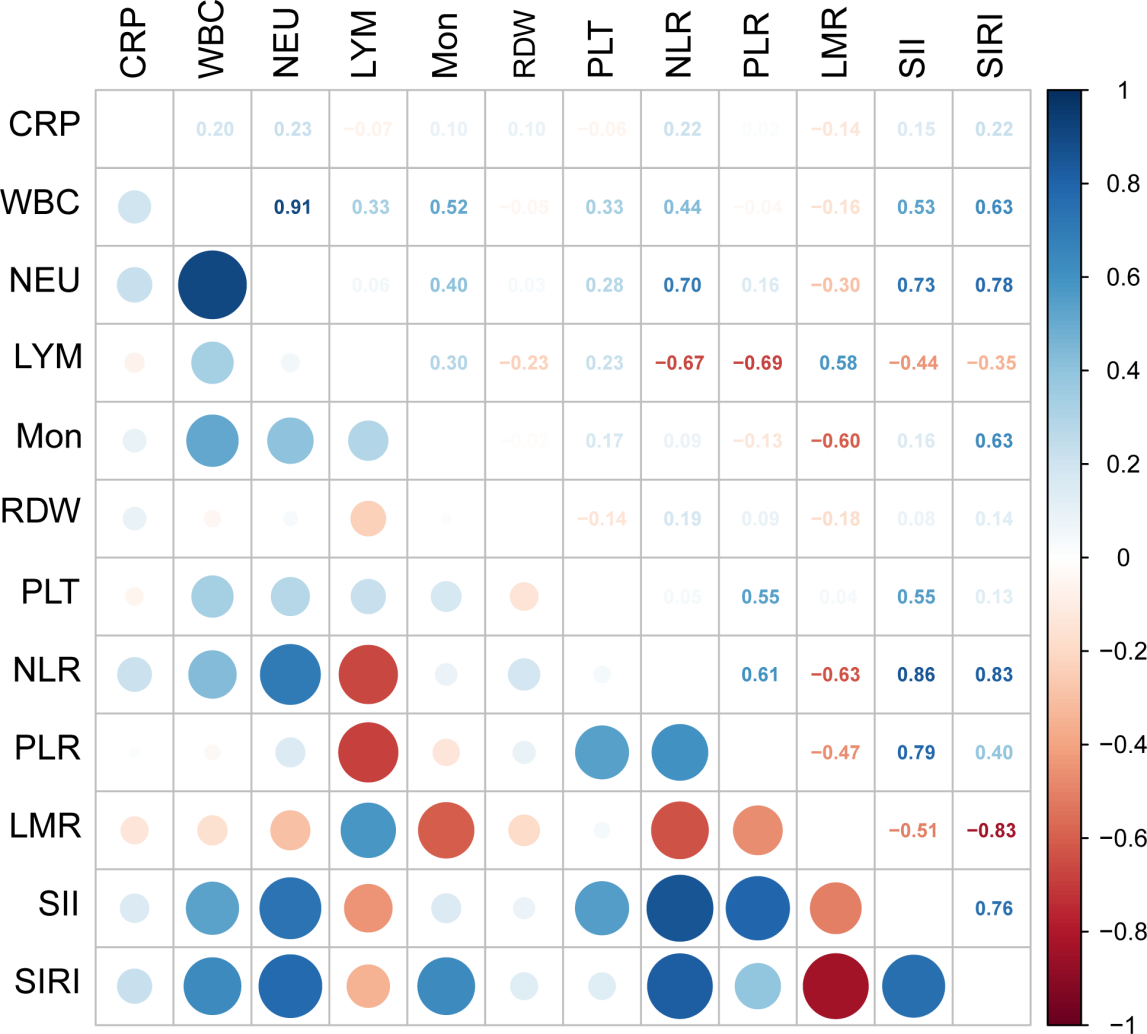
Pairwise pearson correlation coefficients among the 12 inflammatory markers. Blue indicates positive correlation, and red indicates negative correlation. Darker colors are associated with stronger correlation coefficients. CRP, C-reactive protein; WBC, white blood cell; NEU, neutrophils; LYM, lymphocyte; Mon, monocyte; RDW, red cell distribution width; PLT, platelet; NLR, neutrophil-to-lymphocyte ratio; PLR, platelet-to-lymphocyte ratio; LMR, lymphocyte-to-monocyte ratio monocyte; SII, systemic immune-inflammation index; SIRI, systemic inflammatory response index.

The Pearson correlation method was adopted to calculate correlation coefficients for the 12 inflammatory markers, which showed a high correlation among the inflammatory markers (**Figure 3**).

**Figure 4:**
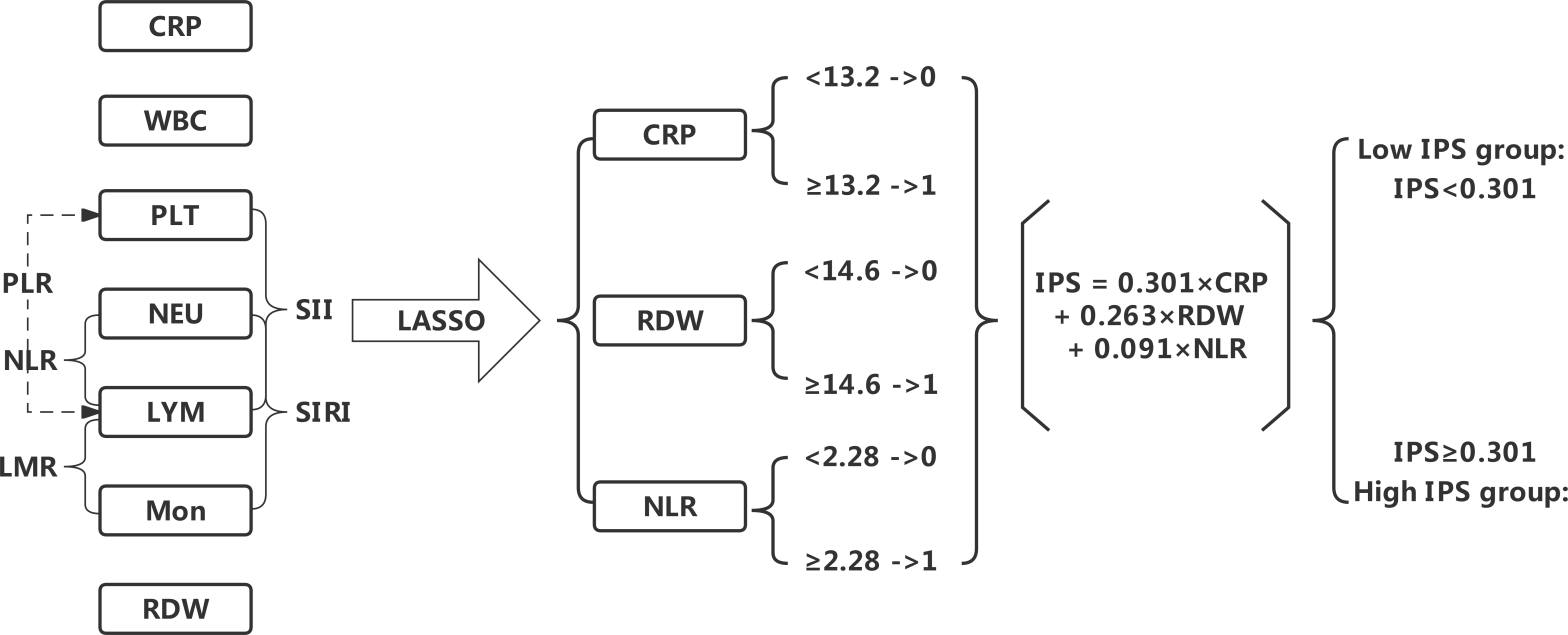
Flow chart for the development of the inflammatory prognostic scoring (IPS).

**Figure 5:**
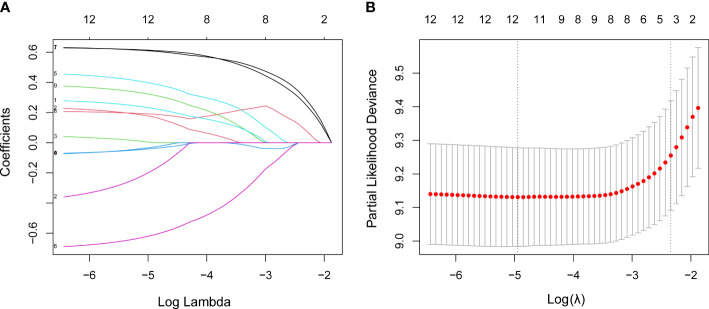
LASSO regression analysis performed feature selection on 12 inflammatory markers using a 5-fold cross-validation method. **(A)** Coefficient profile diagram. Each curve in the figure represents the change trajectory of each independent variable coefficient. The ordinate is the value of the coefficient, and the abscissa is the number of non-zero coefficients in the model. With the constant value of the penalty parameter lambda increases, the final variable coefficient gradually approaches 0. **(B)** The left dashed line corresponds to the λ value with the smallest mean square error, and the right dashed line corresponds to the λ value of the simplest model within a variance range of the minimum λ value.

The LASSO analysis was used for data dimensionality reduction and variable selection while constructing the IPS system (**Figure 4**). Among the 12 candidate inflammatory biomarkers, 3 were non-zero coefficients, namely CRP, RDW and NLR, and the optimal λ value was equal to 0.096, log (λ) = - 2.341 (**Figures 5A, B**). The IPS of patient with AHF was calculated based on the corresponding regression coefficient of LASSO, and the calculation was as follows: IPS = 0.301×CRP + 0.263×RDW + 0.091×NLR.

**Figure 6:**
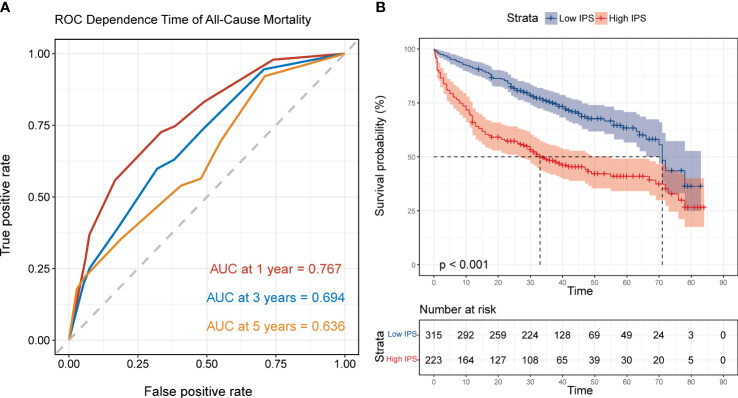
The predictive values of inflammatory prognostic scoring (IPS). **(A)** Time-dependent ROC curves showing the area under the curve of IPS predicting all-cause mortality at 1, 3 and 5 years in AHF patients; **(B)** Kaplan-Meier survival curve of IPS for all-cause mortality in AHF patients, grouped by ROC optimal cut-off values. Low IPS: <0.301 points, n=315; and High IPS: ≥0.301 points, n=223.

The results of the time-dependent ROC curve regarding IPS for all-cause mortality showed that the AUC at 1 year, 3 years and 5 years was 0.767 (0.720-0.817), 0.694 (0.642-0.742) and 0.636 (0.560-0.711) respectively; and the optimal cut-off value of IPS to predict the 3-year all-cause mortality in AHF was 0.301 points (**Figure 6A**). Similarly, the patients were divided into low-IPS group (<0.301 points, n=315) and high-IPS group (≥0.301 points, n=223) by the optimal cut-off value. The Kaplan-Meier survival curve showed that the all-cause mortality in the high IPS group was significantly higher than that in the low IPS group (*P* log-rank <0.001, **Figure 6B**).

### Prognostic association of inflammatory markers with all-cause mortality of AHF

In the multivariate analysis, the variables with *P*<0.10 in the univariate analysis (except 12 inflammatory markers) were included in the multivariate COX regression model. The results of the stepwise regression analysis showed that age (HR=1.023 [1.014-1.033]; *P*<0.001), female (HR=1.487 [1.140-1.940]; *P*=0.003), mean arterial pressure (MAP, HR=0.980 [0.970-0.989]; *P*<0.001), BUN (HR=1.010 [1.003-1.018]; *P*=0.005) and Log2 NT-proBNP (HR=1.267 [1.145-1.401]; *P*<0.001) were independent risk factors for all-cause mortality of patients with AHF (**Table 3**).

**Table 3:** Univariate and multivariate stepwise Cox regression analysis of all-cause mortality in patients with AHF.

	Univariate analysis		Multivariate analysis	
	HR (95% CI)	*P* value	HR (95% CI)	*P* value
Age, years	1.026 (1.016-1.035)	<0.001	1.023 (1.014-1.033)	<0.001
Female, %	1.691 (1.300-2.199)	<0.001	1.487 (1.140-1.940)	0.003
NYHA functional class, %		0.002		
II	1 [Reference]			
III	1.706 (1.118-2.603)	0.013		
IV	2.214 (1.416-3.461)	<0.001		
Average heart rate, bpm	0.991 (0.983-1.000)	0.047		
Systolic BP, mmHg	0.991 (0.985-0.997)	0.005		
Diastolic BP, mmHg	0.980 (0.970-0.989)	<0.001		
MAP, mmHg	0.981 (0.972-0.990)	<0.001	0.980 (0.970-0.989)	<0.001
Hemoglobin, g/L	0.988 (0.983-0.994)	<0.001		
Triglycerides, mmol/L	0.686 (0.541-0.869)	0.002		
BUN, mmol/L	1.016 (1.010-1.022)	<0.001	1.010 (1.003-1.018)	0.005
eGFR, mL/(min·1.73 m2)	0.985 (0.980-0.990)	<0.001		
Log2 NT-proBNP, ng/L	1.300 (1.178-1.435)	<0.001	1.267 (1.145-1.401)	<0.001
LVEF, %	1.009 (1.000-1.018)	0.051		

LVEF, left ventricular ejection fraction; NYHA, New York Heart Association; BP, blood pressure; MAP, mean arterial pressure; BUN, blood urea nitrogen; eGFR, estimated glomerular filtration rate; NT-pro BNP, N-Terminal pro-brain natriuretic peptide.

Further COX regression analysis regarding the inflammatory markers showed that high-IPS (HR=1.688 [1.280-2.228]), CRP (HR=1.868 [1.418-2.461]), WBC (HR=1.496 [1.136-1.972]), NEU (HR=1.469 [1.108-1.947]), Mon (HR=1.565 [1.195-2.049]), RDW (HR=1.695 [1.296-2.218]), NLR (HR=1.483 [1.095-2.008]), LMR (HR=1.452 [1.087-1.941]), SII (HR=1.424 [1.043-1.943]) and SIRI (HR=1.387 [1.061- 1.814]) were independently associated with an increased risk of all-cause mortality in patients with AHF, while LYM, PLT and PLR were not associated with the prognosis in patients with AHF after a complete adjustment of the basic prognostic model (age, sex, MAP, BUN, and Log2 NT-proBNP, **Table 4**).

**Table 4:** Association of inflammatory biomarkers with all-cause mortality in patients with AHF.

	Univariate analysis		Multivariate analysis †	
	HR (95% CI)	*P* value	HR (95% CI)	*P* value
High IPS	2.227 (1.713-2.896)	<0.001	1.688 (1.280-2.228)	<0.001
CRP≥13.2, mg/L	2.163 (1.658-2.822)	<0.001	1.868 (1.418-2.461)	<0.001
WBC≥7.10, 10^9^/L	1.376 (1.060-1.786)	0.016	1.496 (1.136-1.972)	0.004
NEU≥5.40, 10^9^/L	1.510 (1.153-1.978)	0.003	1.469 (1.108-1.947)	0.008
LYM<1.76, 10^9^/L	1.595 (1.201-2.117)	0.001	1.256 (0.938-1.680)	0.125
Mon≥0.43, 10^9^/L	1.462 (1.122-1.906)	0.005	1.565 (1.195-2.049)	0.001
RDW≥14.6, %	2.071 (1.594-2.690)	<0.001	1.695 (1.296-2.218)	<0.001
PLT<123, 10^9^/L	1.697 (1.282-2.246)	<0.001	1.298 (0.973-1.731)	0.076
NLR≥2.28	1.957 (1.463-2.619)	<0.001	1.483 (1.095-2.008)	0.011
PLR≥99.66	1.352 (1.040-1.757)	0.024	1.254 (0.959-1.640)	0.098
LMR<3.97	1.770 (1.335-2.348)	<0.001	1.452 (1.087-1.941)	0.012
SII≥310.73, 10^9^/L	1.576 (1.159-2.145)	0.004	1.424 (1.043-1.943)	0.026
SIRI≥1.51, 10^9^/L	1.628 (1.254-2.113)	<0.001	1.387 (1.061-1.814)	0.017

†Model was adjusted for age, sex, MAP, BUN and Log2 NT-proBNP.

IPS, inflammatory prognostic scoring; CRP, C-reactive protein; WBC, white blood cell; NEU, neutrophils; LYM, lymphocyte; Mon, monocyte; RDW, red blood cell distribution width; PLT, platelet; NLR, neutrophil-to-lymphocyte ratio; PLR, platelet to lymphocyte ratio; LMR, lymphocyte-to-monocyte ratio; SII, systemic inflammation index; SIRI, systemic inflammatory response index.

### Improvement of inflammatory markers in the basic model of AHF prognosis

Time-dependent ROC (1 year, 3 years and 5 years) was used to evaluate the improvement degree of inflammatory biomarkers in the baseline model of AHF prognosis (**Table 5**). The AUC of the basic prognostic model (age, sex, mean arterial pressure, blood urea nitrogen and Log2 NT-proBNP) was 1-year (AUC=0.739 [0.682-0.795]), 3-year (AUC=0.738 [0.690-0.787]), and 5-year (AUC=0.671 [0.600-0.742]) respectively.

**Table 5:** Improvement of the prognostic model by the addition of 12 inflammatory biomarkers to the base model, respectively.

	1 year AUC	*P* value	3 years AUC	*P* value	5 years AUC	*P* value
Model †	0.739 (0.682-0.795)		0.738 (0.690-0.787)		0.671 (0.600-0.742)	
+ High IPS	0.803 (0.754-0.851)	<0.001	0.764 (0.717-0.810)	0.042	0.686 (0.613-0.759)	0.459
+ CRP≥13.2, mg/L	0.781 (0.729-0.833)	0.002	0.745 (0.697-0.792)	0.569	0.670 (59.61-74.39)	0.954
+ WBC≥7.10, 10^9^/L	0.748 (0.693-0.803)	0.279	0.753 (0.706-0.800)	0.060	0.675 (0.602-0.747)	0.762
+ NEU≥5.40, 10^9^/L	0.746 (0.690-0.802)	0.384	0.753 (0.705-0.800)	0.051	0.681 (0.608-0.753)	0.369
+ LYM<1.76, 10^9^/L	0.742 (0.686-0.798)	0.148	0.739 (0.690-0.787)	0.813	0.672 (0.601-0.744)	0.683
+ Mon≥0.43, 10^9^/L	0.750 (0.695-0.804)	0.238	0.751 (0.704-0.798)	0.154	0.672 (0.600-0.743)	0.969
+ RDW≥14.6, %	0.762 (0.709-0.816)	0.036	0.753 (0.706-0.801)	0.134	0.688 (0.618-0.759)	0.266
+ PLT<123, 10^9^/L	0.741 (0.685-0.797)	0.688	0.748 (0.700-0.795)	0.053	0.674 (0.602-0.746)	0.689
+ NLR≥2.28	0.753 (0.698-0.808)	0.064	0.750 (0.702-0.797)	0.111	0.679 (0.607-0.751)	0.463
+ PLR≥99.66	0.751 (0.695-0.807)	0.010	0.745 (0.697-0.793)	<0.001	0.671 (0.600-0.742)	<0.001
+ LMR<3.97	0.750 (0.695-0.804)	0.143	0.741 (0.693-0.789)	0.680	0.662 (0.590-0.735)	0.454
+ SII≥310.73, 10^9^/L	0.744 (0.688-0.799)	0.471	0.744 (0.696-0.792)	0.363	0.673 (0.601-0.745)	0.856
+ SIRI≥1.51, 10^9^/L	0.752 (0.697-0.807)	0.080	0.741 (0.693-0.789)	0.703	0.664 (0.591-0.736)	0.509

†The model included age, sex, MAP, BUN and Log2 NT-proBNP.

IPS, inflammatory prognostic scoring; CRP, C-reactive protein; WBC, white blood cell; NEU, neutrophils; LYM, lymphocyte; Mon, monocyte; RDW, red blood cell distribution width; PLT, platelet; NLR, neutrophil-to-lymphocyte ratio; PLR, platelet to lymphocyte ratio; LMR, lymphocyte-to-monocyte ratio; SII, systemic inflammation index; SIRI, systemic inflammatory response index.

Further analyses with the IPS and 12 inflammatory markers showed that the AUC was improved most significantly in the basic model addition to IPS (1 year: AUC= 0.803 [0.754-0.851], *P <*0.001; 3 years: AUC=0.764, [0.717-0.810], *P*=0.042; 5 years: AUC=0.686 [0.613-0.759], *P*=0.459). The 1-year risk prediction ability was significantly improved with CRP, RDW and PLT, while other inflammatory markers did not show significant improvement in the risk prediction ability of the AHF basic model.

### Importance of inflammatory markers in predicting all-cause mortality

**Figure 7:**
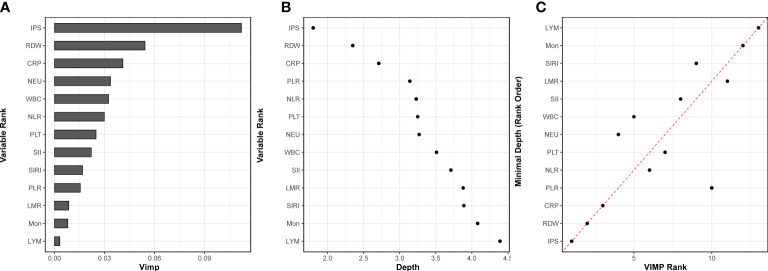
Importance from random survival forest analysis for all-cause mortality in acute heart failure patients. **(A)** variable importance (VIMP) is the difference in validation set prediction error before and after the permutation of variables, the larger values of VIMP indicate the more predictive of the variable. **(B)** minimal depth (MD) indicates the impact of the variable on the prediction, the lower values of MD indicate that variable has stronger predictive value. **(C)** Comparison of the VIMP ranking and MD. The farther the points are from the diagonal dash-line, the more the discrepancy between measures. IPS, inflammatory prognostic scoring; CRP, C-reactive protein; WBC, white blood cell; NEU, neutrophils; LYM, lymphocyte; Mon, monocyte; RDW, red cell distribution width; PLT, platelet; NLR, neutrophil-to-lymphocyte ratio; PLR, platelet-to-lymphocyte ratio; LMR, lymphocyte-to-monocyte ratio monocyte; SII, systemic immune-inflammation index; SIRI, systemic inflammatory response index.

RSF analyses with VIMP and MD were further plotted to validate their predictive value for all-cause mortality in patients with AHF. Among IPS and all the 12 inflammatory markers, IPS was the most predictive for all-cause mortality in patients with AHF, according to both VIMP and MD values (**Figures 7A, B**). The VIMP and MD were consistent in evaluating the relative importance of IPS and predicting the risk of all-cause mortality (**Figure 7C**).

## Discussion

This study was a prospective cohort study on 538 patients with AHF to analyze the correlation between 12 inflammatory biomarkers and all-cause mortality in patients with AHF. Based on the optimal cut-off and LASSO analysis, an IPS system, which included CRP, RDW and NLR, was constructed. IPS remained an independent prognostic predictor for patients with AHF in multivariate COX regression analysis after adjusting the significant markers. Furthermore, IPS improved the prediction values most significantly in the ROC, which was the most importance variable among the inflammatory markers in the random survival forest.

Systemic inflammation can promote the activation of related cytokines and the migration of monocytes to myocardial tissues (30), which could lead to myocardial interstitial fibrosis and ventricular remodeling (5, 31) in patients with HF. Cytokines secreted by monocytes or released due to the hyperemia and activation of the hypothalamic-pituitary-adrenal axis could induce lymphocyte apoptosis and affect the functions of circulating lymphocytes (32). At the same time, pro-inflammatory cytokines, lipopolysaccharides and hypoxic signals prolong neutrophil apoptosis (33), and an increased release of granulocytes or granulocyte-macrophage colony-stimulating factors also contributes to the prolongation of the lifespan of neutrophils and granulocytes (34). Therefore, these biological functions of WBC, specifically NEU, are one of the major factors of cardiac dysfunctions and AHF *via* the involvement of myocardial tissues and endothelial cells (35).

Given these interdependent pathophysiological pathways, inflammation plays an important role in the pathogenesis and progression of AHF. The elevated level of inflammatory markers is associated with HF severity and prognosis (7, 36, 37). Indexes and parameters derived from CBC for the assessment of inflammatory processes are often useful in the diagnoses and assessments in HF. It has been found through studies that an increased Mon is associated with an increased all-cause mortality in patients with HF and a reduced ejection fraction (HFrEF) as well as HF with preserved ejection fraction (HFpEF) (12, 13). The EVEREST trial showed that higher Mon counts (≥800/μl) were associated with an increased risk of all-cause mortality (HR=1.27 [1.00-1.60]), but not the cardiovascular mortality or hospitalization of HF among patients with HFrEF at a 9.9-month median follow-up (12). In addition, a decreased LYM was associated with an increased short-term mortality and rehospitalization in HF (14, 15). The Pre-RELAX-AHF study showed that patients with AHF with a lower LYM ratio (<13%) during the 60-day and 180-day follow-up were associated with an increased risk of all-cause mortality (HR=1.11 [1.03-1.19]) (14). The same results were observed in another prospective cohort study involving 309 participants, where a low lymphocyte count (<1410/μl) was an independent predictor of long-term mortality in patients with AHF (HR=2.04 [1.06-3.95]) (15). Among patients with myocardial infarction, an increased NEU could be used to predict the development of AHF (38), while both a decreased and an increased PLT were associated with an increased mortality in a “U”-shaped relationship (16). CBC-derived parameters such as NLR, PLR, LMR and SII have also been extensively studied and proven to be highly-sensitive biomarkers of the disease, which could reveal the alterations in the immunological balance due to various pathologies and were shown to be associated with the prognosis in patients with AHF (18–21, 39).

On the other hand, CRP is an upstream acute response marker of inflammation in the liver in response to IL-1 activation *via* IL-6. The increase in CRP is also closely related to the presence of numerous comorbidities such as diabetes, chronic obstructive pulmonary disease, renal failure and peripheral artery disease, which contributes to the progression of HF, negatively impacting the prognosis of patients. An elevated CRP level in patients with HFrEF and HFpEF can be observed in various studies (40, 41). In a study involving 22756 participants from 4 cohorts and a median follow-up of 12 years, CRP was associated with the incident HFrEF in a multivariate-adjusted model (HR=1.19 [1.11-1.28]) (40). The results of a meta-analysis including 19 studies similarly showed that CRP, as a continuous variable, was associated with the incident HFpEF (HR=1.08 [1.00-1.16]), cardiovascular mortality (HR=1.24 [1.04-1.47]) and all-cause mortality (HR=1.06 [1.02-1.06]) in patients with HFpEF (41). Similarly, RDW reflects the degree of erythrocyte variation, which is associated with inflammation, oxidative stress and erythrocyte variation (42). Studies have shown that oxidative stress shortens the lifespan of red blood cells and changes the distribution of red blood cell volume. RDW may reflect the inflammatory response, which is a powerful biomarker for long-term prognosis in patients with AHF (17, 43). In a cohort study including 3231 patients with HF with a median follow-up of 2.9 years, those with RDW in the upper quartile had an increased risk of all-cause mortality, 120% and 114% increased risk of all-cause mortality (HR=2.20 [1.68–2.89]) and cardiovascular mortality (HR=2.14 [1.53–2.98]) respectively, compared to patients with RDW in the lower quartile (43).

Altogether, inflammatory biomarkers play diverse roles in the progression of myocardial fibrosis, persistent systemic inflammation, endothelial injuries or the impairment of left ventricular systolic function, leading to a worsening HF. The elevated circulating level of inflammatory biomarkers is not only a consequence of HF progression but also a cause of AHF progression. It was demonstrated in the current study that RDW, CRP and NLR were the most important inflammatory markers for predicting the poor prognosis in patients with AHF among the 12 inflammatory markers. However, as a non-specific systemic biomarker of inflammation, evaluations using single parameters are easily influenced by numerous factors and disease states due to the complexity and diversity of errors (44). Therefore, risk stratification and mortality prediction in AHF should be enhanced in the implementation of combined markers/parameters. In this study, we performed a LASSO-COX regression to analyze 12 inflammatory biomarkers and eventually selected 3 inflammatory markers (CRP, RDW and NLR) with non-zero coefficients to develop a novel IPS. This method provided for a comprehensive analysis on the predictive value of inflammatory biomarkers while avoiding the effect of multicollinearity problems on the prediction model to some extent.

Currently, several prognostic models are available in clinical practice to assess the prognosis of patients with AHF, including ELAN-HF Score (45), OPTIMIZE-HF Risk Score (46) and GWTG-HF risk score (47). In our study, further integrating these markers as an IPS system remained an independent risk factor for AHF prognosis, which could improve the predicting value of all-cause mortality in patients with AHF in addition to the basic prognostic model (age, sex, MAP, BUN and NT-proBNP), with a model AUC of 0.803 (0.754-0.851; *P*<0.001), 0.764 (0.717-0.810; *P*=0.042) and 0.686 (0.613-0.759; *P*=0.459) at 1 year, 3 years and 5 years respectively, which were higher than that of the predictive models mentioned above. Compared with these models, the IPS constructed based on CRP, RDW and NLR, which were easily collected in clinical practice and easy to use, could be used to accurately quantify the risk of all-cause mortality in patients with AHF, which is important for the individualized risk stratification of patients and an improved prognosis.

The current study also has certain limitations. Firstly, the study was single-center with a relatively small sample size, which needs a larger cohort and an external validation to further verify the results. Secondly, certain novel inflammatory markers (such as interleukin-6 and tumor necrosis factor-alpha) are not included in the analysis due to the accessibility. Thirdly, the fluctuations of inflammatory biomarkers without repeated measurement during discharge or follow-up of patients, which are related to the poor prognosis in AHF, remain to be elucidated. The influence of confounding factors cannot be completely ruled out when interpreting the study results.

## Conclusion

By integrating the clinically accessible inflammatory markers, an inflammatory prognostic scoring (IPS) system was constructed for patients with AHF in the current study. IPS calculated with CRP, RDW and NLR showed that it remained an independent predicator of all-cause mortality in patients with AHF. Further random survival forest analyses demonstrated that IPS had the most important predictive value among the inflammatory markers, suggesting that it has a great potential in serving as a practical tool. In addition, we think that the findings should be further externally validated in large prospective, multicenter cohort study.

## Data availability statement

The original contributions presented in the study are included in the article/Supplementary Material. Further inquiries can be directed to the corresponding authors.

## Ethics statement

The studies involving human participants were reviewed and approved by the Institutional Review and Ethics Board of the First Affiliated Hospital of Nanjing Medical University, Jiangsu Province Hospital. The patients/participants provided their written informed consent to participate in this study.

## Author contributions

XZ: Conceptualization, Methodology, Software, Formal analysis, Writing-original draft, Visualization. IC: Formal analysis, Methodology, Writing-review and editing. FX: Formal analysis, Data curation, Writing-original draft. RG: Project administration, Writing-review and editing. SL: Data curation and Supervision. WY: Project administration, Writing-review and editing. YZ: Project administration, Writing-review and editing. HZ: Conceptualization, Methodology, Writing-review and editing, Supervision. XL: Conceptualization, Methodology, Project administration, Writing-review and editing, Supervision. All authors contributed to the article and approved the submitted version.

## Funding

This study received grant support from the Twelve-Fifth National Key Technology Research & Development Program (2011BAI11B08).

## Conflict of interest

The authors declare that the research was conducted in the absence of any commercial or financial relationships that could be construed as a potential conflict of interest.

## Publisher’s note

All claims expressed in this article are solely those of the authors and do not necessarily represent those of their affiliated organizations, or those of the publisher, the editors and the reviewers. Any product that may be evaluated in this article, or claim that may be made by its manufacturer, is not guaranteed or endorsed by the publisher.

## References

[1] McDonagh TA Metra M Adamo M Gardner RS Baumbach A Böhm M. 2021 ESC guidelines for the diagnosis and treatment of acute and chronic heart failure. Eur Heart J (2021) 42(36):3599–726. doi: 10.1093/eurheartj/ehab368 34447992

[2] Lainščak M Milinković I Polovina M Crespo-Leiro MG Lund LH Anker SD. Sex- and age-related differences in the management and outcomes of chronic heart failure: An analysis of patients from the ESC HFA EORP heart failure long-term registry. Eur J Heart Fail (2020) 22(1):92–102. doi: 10.1002/ejhf.1645 31863522

[3] Hao G Wang X Chen Z Zhang L Zhang Y Wei B. Prevalence of heart failure and left ventricular dysfunction in China: The China hypertension survey, 2012-2015. Eur J Heart Fail (2019) 21(11):1329–37. doi: 10.1002/ejhf.1629 31746111

[4] Li L Liu R Jiang C Du X Huffman MD Lam CSP. Assessing the evidence-practice gap for heart failure in China: The heart failure registry of patient outcomes (HERO) study design and baseline characteristics. Eur J Heart Fail (2020) 22(4):646–60. doi: 10.1002/ejhf.1630 31820513

[5] Chioncel O Mebazaa A Harjola VP Coats AJ Piepoli MF Crespo-Leiro MG. Clinical phenotypes and outcome of patients hospitalized for acute heart failure: The ESC heart failure long-term registry. Eur J Heart Fail (2017) 19(10):1242–54. doi: 10.1002/ejhf.890 28463462

[6] Mann DL. The emerging role of innate immunity in the heart and vascular system: For whom the cell tolls. Circ Res (2011) 108(9):1133–45. doi: 10.1161/circresaha.110.226936 PMC3084988 21527743

[7] Murphy SP Kakkar R McCarthy CP Januzzi JL Jr. Inflammation in heart failure: JACC state-of-the-Art review. J Am Coll Cardiol (2020) 75(11):1324–40. doi: 10.1016/j.jacc.2020.01.014 32192660

[8] Zhang Y Bauersachs J Langer HF. Immune mechanisms in heart failure. Eur J Heart Fail (2017) 19(11):1379–89. doi: 10.1002/ejhf.942 28891154

[9] Magnussen C Blankenberg S. Biomarkers for heart failure: small molecules with high clinical relevance. J Intern Med (2018) 283(6):530–43. doi: 10.1111/joim.12756 29682806

[10] Passino C Barison A Vergaro G Gabutti A Borrelli C Emdin M. Markers of fibrosis, inflammation, and remodeling pathways in heart failure. Clin Chim Acta (2015) 443:29–38. doi: 10.1016/j.cca.2014.09.006 25218738

[11] Pearson TA Mensah GA Alexander RW Anderson JL Cannon RO 3rd Criqui M. Markers of inflammation and cardiovascular disease: application to clinical and public health practice: A statement for healthcare professionals from the centers for disease control and prevention and the American heart association. Circulation (2003) 107(3):499–511. doi: 10.1161/01.cir.0000052939.59093.45 12551878

[12] Greene SJ Harinstein ME Vaduganathan M Subačius H Konstam MA Zannad F. Prognostic value of monocyte count in patients hospitalized for heart failure with reduced ejection fraction (from the EVEREST trial). Am J Cardiol (2012) 110(11):1657–62. doi: 10.1016/j.amjcard.2012.07.035 22917555

[13] Shantsila E Bialiuk N Navitski D Pyrochkin A Gill PS Pyrochkin V. Blood leukocytes in heart failure with preserved ejection fraction: Impact on prognosis. Int J Cardiol (2012) 155(2):337–8. doi: 10.1016/j.ijcard.2011.12.048 22227252

[14] Milo-Cotter O Teerlink JR Metra M Felker GM Ponikowski P Voors AA. Low lymphocyte ratio as a novel prognostic factor in acute heart failure: Results from the pre-RELAX-AHF study. Cardiology (2010) 117(3):190–6. doi: 10.1159/000321416 21088400

[15] Carubelli V Bonadei I Castrini AI Gorga E Ravera A Lombardi C. Prognostic value of the absolute lymphocyte count in patients admitted for acute heart failure. J Cardiovasc Med (Hagerstown) (2017) 18(11):859–65. doi: 10.2459/jcm.0000000000000428 27541359

[16] Song PS Ahn KT Jeong JO Jeon KH Song YB Gwon HC. Association of baseline platelet count with all-cause mortality after acute myocardial infarction. Eur Heart J Acute Cardiovasc Care (2020). doi: 10.1177/2048872620925257 32403936

[17] Melchio R Rinaldi G Testa E Giraudo A Serraino C Bracco C. Red cell distribution width predicts mid-term prognosis in patients hospitalized with acute heart failure: The RDW in acute heart failure (RE-AHF) study. Intern Emerg Med (2019) 14(2):239–47. doi: 10.1007/s11739-018-1958-z 30276661

[18] Curran FM Bhalraam U Mohan M Singh JS Anker SD Dickstein K. Neutrophil-to-lymphocyte ratio and outcomes in patients with new-onset or worsening heart failure with reduced and preserved ejection fraction. ESC Heart Fail (2021) 8(4):3168–79. doi: 10.1002/ehf2.13424 PMC8318449 33998162

[19] Angkananard T Inthanoo T Sricholwattana S Rattanajaruskul N Wongsoasu A Roongsangmanoon W. The predictive role of neutrophil-to-Lymphocyte ratio (NLR) and mean platelet volume-to-Lymphocyte ratio (MPVLR) for cardiovascular events in adult patients with acute heart failure. Mediators Inflammation (2021) 2021:6889733. doi: 10.1155/2021/6889733 PMC8523242 34671226

[20] Heidarpour M Bashiri S Vakhshoori M Heshmat-Ghahdarijani K Khanizadeh F Ferdowsian S. The association between platelet-to-lymphocyte ratio with mortality among patients suffering from acute decompensated heart failure. BMC Cardiovasc Disord (2021) 21(1):454. doi: 10.1186/s12872-021-02260-7 34537010 PMC8449504

[21] Tang Y Zeng X Feng Y Chen Q Liu Z Luo H. Association of systemic immune-inflammation index with short-term mortality of congestive heart failure: A retrospective cohort study. Front Cardiovasc Med (2021) 8:753133. doi: 10.3389/fcvm.2021.753133 34869661 PMC8632819

[22] Yamaguchi S Abe M Arakaki T Arasaki O Shimabukuro M. Incremental prognostic value of platelet count in patients with acute heart failure - a retrospective observational study. Circ J (2019) 83(3):576–83. doi: 10.1253/circj.CJ-18-0961 30606941

[23] Heart Failure Group of Chinese Society of Cardiology of Chinese Medical Association. [Chinese guidelines for the diagnosis and treatment of heart failure 2014]. Chinese Journal of Cardiology (2014) 42(2):98–122. doi: 10.3760/cma.j.issn.0253-3758.2014.02.004 24735621

[24] Matsushita K Mahmoodi BK Woodward M Emberson JR Jafar TH Jee SH. Comparison of risk prediction using the CKD-EPI equation and the MDRD study equation for estimated glomerular filtration rate. Jama (2012) 307(18):1941–51. doi: 10.1001/jama.2012.3954 PMC3837430 22570462

[25] Le NQK Ho QT. Deep transformers and convolutional neural network in identifying DNA N6-methyladenine sites in cross-species genomes. Methods (2022) 204:199–206. doi: 10.1016/j.ymeth.2021.12.004 34915158

[26] Tng SS Le NQK Yeh HY Chua MCH. Improved prediction model of protein lysine crotonylation sites using bidirectional recurrent neural networks. J Proteome Res (2022) 21(1):265–73. doi: 10.1021/acs.jproteome.1c00848 34812044

[27] Breiman L. Random forests. Mach Learn (2001) 45(1):5–32. doi: 10.1023/A:1010933404324

[28] Mogensen UB Ishwaran H Gerds TA. Evaluating random forests for survival analysis using prediction error curves. J Stat Softw (2012) 50(11):1–23. doi: 10.18637/jss.v050.i11 25317082 PMC4194196

[29] Komajda M Hanon O Hochadel M Lopez-Sendon JL Follath F Ponikowski P. Contemporary management of octogenarians hospitalized for heart failure in Europe: Euro heart failure survey II. Eur Heart J (2009) 30(4):478–86. doi: 10.1093/eurheartj/ehn539 19106198

[30] Vaduganathan M Greene SJ Butler J Sabbah HN Shantsila E Lip GY. The immunological axis in heart failure: Importance of the leukocyte differential. Heart Fail Rev (2013) 18(6):835–45. doi: 10.1007/s10741-012-9352-9 23054221

[31] Swirski FK Nahrendorf M Etzrodt M Wildgruber M Cortez-Retamozo V Panizzi P. *et al*: Identification of splenic reservoir monocytes and their deployment to inflammatory sites. Science (2009) 325(5940):612–6. doi: 10.1126/science.1175202 PMC2803111 19644120

[32] Maisel AS Knowlton KU Fowler P Rearden A Ziegler MG Motulsky HJ. Adrenergic control of circulating lymphocyte subpopulations. Effects of congestive heart failure, dynamic exercise, and terbutaline treatment. J Clin Invest (1990) 85(2):462–7. doi: 10.1172/jci114460 PMC296446 2153706

[33] Theofilis P Sagris M Oikonomou E Antonopoulos AS Siasos G Tsioufis C. Inflammatory mechanisms contributing to endothelial dysfunction. Biomedicines (2021) 9(7):781. doi: 10.3390/biomedicines9070781 34356845 PMC8301477

[34] Raffaghello L Bianchi G Bertolotto M Montecucco F Busca A Dallegri F. Human mesenchymal stem cells inhibit neutrophil apoptosis: A model for neutrophil preservation in the bone marrow niche. Stem Cells (2008) 26(1):151–62. doi: 10.1634/stemcells.2007-0416 17932421

[35] Tracchi I Ghigliotti G Mura M Garibaldi S Spallarossa P Barisione C. Increased neutrophil lifespan in patients with congestive heart failure. Eur J Heart Fail (2009) 11(4):378–85. doi: 10.1093/eurjhf/hfp031 19276127

[36] Hage C Michaëlsson E Linde C Donal E Daubert JC Gan LM. Inflammatory biomarkers predict heart failure severity and prognosis in patients with heart failure with preserved ejection fraction: A holistic proteomic approach. Circ Cardiovasc Genet (2017) 10(1):e001633. doi: 10.1161/circgenetics.116.001633 28100627

[37] Bouras G Giannopoulos G Hatzis G Alexopoulos D Leventopoulos G Deftereos S. Inflammation and chronic heart failure: From biomarkers to novel anti-inflammatory therapeutic strategies. Med Chem (2014) 10(7):682–99. doi: 10.2174/1573406410666140318113325 25102199

[38] Kyne L Hausdorff JM Knight E Dukas L Azhar G Wei JY. Neutrophilia and congestive heart failure after acute myocardial infarction. Am Heart J (2000) 139(1 Pt 1):94–100. doi: 10.1016/s0002-8703(00)90314-4 10618568

[39] Silva N Bettencourt P Guimarães JT. The lymphocyte-to-monocyte ratio: an added value for death prediction in heart failure. Nutr Metab Cardiovasc Dis (2015) 25(11):1033–40. doi: 10.1016/j.numecd.2015.07.004 26482565

[40] de Boer RA Nayor M deFilippi CR Enserro D Bhambhani V Kizer JR. Association of cardiovascular biomarkers with incident heart failure with preserved and reduced ejection fraction. JAMA Cardiol (2018) 3(3):215–24. doi: 10.1001/jamacardio.2017.4987 PMC5862778 29322198

[41] Lakhani I Wong MV Hung JKF Gong M Waleed KB Xia Y. Diagnostic and prognostic value of serum c-reactive protein in heart failure with preserved ejection fraction: A systematic review and meta-analysis. Heart Fail Rev (2021) 26(5):1141–50. doi: 10.1007/s10741-020-09927-x PMC8310477 32030562

[42] Salvagno GL Sanchis-Gomar F Picanza A Lippi G. Red blood cell distribution width: A simple parameter with multiple clinical applications. Crit Rev Clin Lab Sci (2015) 52(2):86–105. doi: 10.3109/10408363.2014.992064 25535770

[43] Liang L Huang L Zhao X Zhao L Tian P Huang B. Prognostic value of RDW alone and in combination with NT-proBNP in patients with heart failure. Clin Cardiol (2022) 45(7):802–13. doi: 10.1002/clc.23850 PMC9286336 35621296

[44] Williams FM. Biomarkers: in combination they may do better. Arthritis Res Ther (2009) 11(5):130. doi: 10.1186/ar2839 19886980 PMC2787279

[45] Salah K Kok WE Eurlings LW Bettencourt P Pimenta JM Metra M. A novel discharge risk model for patients hospitalised for acute decompensated heart failure incorporating n-terminal pro-b-type natriuretic peptide levels: a European coLlaboration on acute decompeNsated heart failure: ELAN-HF score. Heart (2014) 100(2):115–25. doi: 10.1136/heartjnl-2013-303632 24179162

[46] Abraham WT Fonarow GC Albert NM Stough WG Gheorghiade M Greenberg BH. Predictors of in-hospital mortality in patients hospitalized for heart failure: Insights from the organized program to initiate lifesaving treatment in hospitalized patients with heart failure (OPTIMIZE-HF). J Am Coll Cardiol (2008) 52(5):347–56. doi: 10.1016/j.jacc.2008.04.028 18652942

[47] Peterson PN Rumsfeld JS Liang L Albert NM Hernandez AF Peterson ED. A validated risk score for in-hospital mortality in patients with heart failure from the American heart association get with the guidelines program. Circ Cardiovasc Qual Outcomes (2010) 3(1):25–32. doi: 10.1161/circoutcomes.109.854877 20123668

